# Metabolic reprogramming of cancer-associated fibroblasts and its effect on cancer cell reprogramming

**DOI:** 10.7150/thno.62378

**Published:** 2021-07-13

**Authors:** Zhenzhen Li, Chanjun Sun, Zhihai Qin

**Affiliations:** Medical Research Center, The First Affiliated Hospital of Zhengzhou University, Zhengzhou University, Zhengzhou, Henan 450052, China

**Keywords:** Cancer-associated fibroblasts, Cancer, Metabolic reprogramming, Tumor microenvironment

## Abstract

Cancer cells are well-known for adapting their metabolism to maintain high proliferation rates and survive in unfavorable environments with low oxygen and nutritional deficiency. Metabolic reprogramming most commonly arises from the tumor microenvironment (TME). The events of metabolic pathways include the Warburg effect, shift in Krebs cycle metabolites, and increase rate of oxidative phosphorylation that provides the energy for the development and invasion of cancer cells. The TME and shift in tumor metabolism shows a close relationship through bidirectional signaling pathways between the stromal and tumor cells. Cancer-associated fibroblasts (CAFs) are the main type of stromal cells in the TME and consist of a heterogeneous and plastic population that play key roles in tumor growth and metastatic capacity. Emerging evidence suggests that CAFs act as major regulators in shaping tumor metabolism especially through the dysregulation of several metabolic pathways, including glucose, amino acid, and lipid metabolism. The arrangement of these metabolic switches is believed to shape distinct CAF behavior and change tumor cell behavior by the CAFs. The crosstalk between cancer cells and CAFs is associated with cell metabolic reprogramming that contributes to cancer cell growth, progression, and evasion from cancer therapies. But the mechanism and process of this interaction remain unclear. This review aimed to highlight the metabolic couplings between tumor cells and CAFs. We reviewed the recent literature supporting an important role of CAFs in the regulation of cancer cell metabolism, and the relevant pathways, which may serve as targets for therapeutic interventions.

## Introduction

The most prominent biological characteristic of tumor cells is uncontrolled and rapid proliferation. The division of one cell into two requires the doubling of substances such as nucleic acids, amino acids, lipids, and carbohydrates and is the most important metabolic requirement of rapidly proliferating tumor cells, which distinguishes these cells from the vast majority of normal non-cancerous cells. The rapidly proliferating cells must alter their metabolic behavior to achieve the conversion of the highest possible amount of available nutrients into biomass energy, which requires a delicate balance among ATP production, provision of the reducing equivalents, and the synthesis of various biomolecules. In response to factors such as the harsh tumor microenvironment (TME), tumor cells undergo adaptive changes in their metabolic properties, a process referred to as metabolic reprogramming 1. Metabolic reprogramming, in general, is a hallmark of cancer cells 2, and increasing evidence suggests that metabolism plays a central role in tumorigenesis 3. The metabolic abnormalities occurring in tumor cells are not simple alterations of a single metabolic pathway but are rather subversive alterations in the entire cellular network of metabolism. Metabolic reprogramming ensures that the limited nutrients and/or intermediate metabolites are directed toward various biosynthetic pathways that are required to support the energy-demanding anabolic processes for tumor cell proliferation 3. Besides occurring in tumor cells, metabolic reprogramming has also been reported to occur in the TME 4. Metabolic reprogramming of the TME is also considered one of the hallmarks of cancer and represents an important branch of tumor research.

TME, consisting of blood and lymphatic tumor vessels, extracellular matrix (ECM), and non-cancer stromal cells, such as cancer-associated fibroblasts (CAFs), modulates cancer cell growth, progression, and evasion from cancer therapies 5. Especially, CAFs are a major component of the TME 6, 7. CAFs display an activated phenotype; they get larger than their normal counterparts, become spindle-shaped, and show the presence of stress fibers. This phenotype is transiently observed in normal fibroblasts during wound healing. In contrast, CAFs seem to be constantly activated and unable to revert to a quiescent phenotype 8. Moreover, the CAF genome has been reported to undergo epigenetic reprogramming; most studies have not found mutations in CAFs 9.

Associations between CAFs and cancer cells support tumorigenesis in various ways. Besides being a major producer of ECM components, such as collagen and laminins, and remodeling enzymes 10, CAFs can secrete a variety of pro-inflammatory mediators (e.g., interferon-γ, C-X-C motif chemokine ligand 12 (CXCL12), tumor necrosis factor (TNF)-α and several interleukins (ILs)) 11, 12, and growth factors (e.g., transforming growth factor β (TGF-β), fibroblast growth factor (FGF)) 10, as well as, pro-angiogenic factors like vascular endothelial factor (VEGF) and endothelial growth factor (EGF) 13. These functions of CAFs display pro-tumorigenic effects leading to tumor progression. Many reviews have substantially described the interaction of CAFs and cancer cells 14-16. However, recent evidence supports the critical role of CAFs as regulators of important metabolic processes in cancer 17-20. These metabolism-related effects may be specific to fibroblasts in the TME and may be an adaptive mechanism to meet the metabolic demands of the rapidly proliferating tumor cells.

To date, several mechanistic studies have shown the importance of this bioenergetic coupling of tumor and stroma. Therefore, in this review, we focused on the current understanding of the mechanism of cancer cell/CAF metabolic interactions and discussed in detail the activation of different metabolic pathways resulting from these interactions. Detailed metabolic analyses on the tumor cell/CAF crosstalk, including *in vitro* and *in vivo* modeling approaches, have been presented and assessed. Finally, we discussed how reprogrammed CAFs might affect tumor cell metabolic reprogramming and focused on the potential therapeutic strategies targeting molecules involved in the metabolic reprogramming of CAFs.

## Tumor cell/CAF interaction driven by the metabolic reprogramming of fibroblasts

Under physiological conditions, fibroblasts regulate the turnover of ECM, control tissue homeostasis, and participate in wound healing and senescence 5. On the other hand, in solid tumors, normal fibroblasts (NFs) differentiate into CAFs that coevolve with the disorder and alter the biochemical and physical structure of the TME, modifying the behavior of the surrounding stromal and cancer cells 15, 21. Generally, cancer cells highjack CAFs and reprogram their metabolism to perform aerobic glycolysis. CAFs, consequently, secrete metabolites such as pyruvate and lactate, which are taken up by cancer cells and support their metabolic needs as alternative carbon sources.

**Figure 1:**
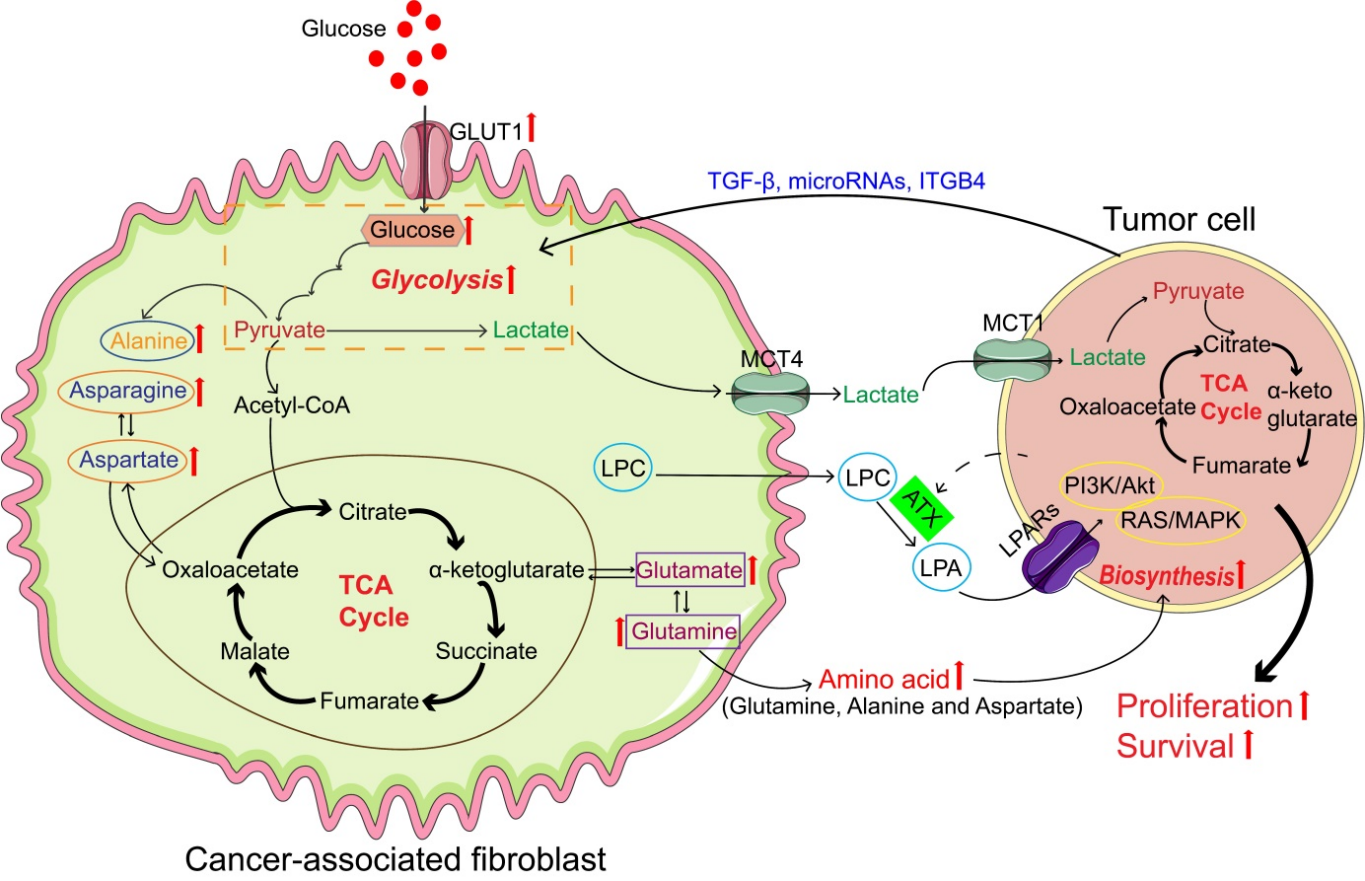
Cancer-associated fibroblasts (CAFs) promote tumor growth through self-metabolic reprogramming: The glycolysis of CAFs was increased in tumor microenvironment, and lactate produced by CAFs was absorbed and utilized by the tumor; CAFs synthesize amino acids through the TCA cycle and amino acids are used by tumor for biosynthesis; In CAFs, lipid metabolism was reprogrammed and LPC was secreted into the microenvironment to promote tumor growth. Abbreviations: GLUT: Glucose transporter; TGF-β: Transforming growth factor-β; ITGB4: Integrin beta 4; MCT1: Monocarboxylate transporter 1; MCT4: Monocarboxylate transporter 4; LPA: Lysophosphatidic acid; LPAR: Lysophosphatidic acid receptor; LPC, lysophosphatidylcholines; TCA, tricarboxylic acid;

CAFs act as active participants in the complex metabolism of tumors rather than just the metabolic co-observers of cancer cells (Figure 1). The arrangement of these metabolic switches is believed to shape distinct CAF behavior and change tumor cell behavior by CAFs. We have summarized the studies on the interaction between CAFs and tumor cells, regarding metabolic reprogramming, in Table 1.

### Glucose Metabolism

Tumor cells perform glycolysis rapidly, even under aerobic conditions, thus increasing glucose uptake and lactate secretion, a phenomenon called the “Warburg effect” or “aerobic glycolysis” 22. CAFs exhibit similar aerobic glycolysis under the influence of tumor cells, which is referred to as the reverse “Warburg Effect” 23. The reverse “Warburg Effect”, occurring in CAFs, is characterized by a shift in the glucose metabolism. In normal cells, glucose is metabolized through oxidative phosphorylation. In contrast to normal fibroblasts, CAFs undergo a significant shift from oxidative phosphorylation to aerobic glycolysis. It has been reported that a reduction in the expression of the α subunit of the isocitrate dehydrogenase 3 complex (IDH3α) is associated with the metabolic switch from oxidative phosphorylation to glycolysis and that overexpression of IDH3α prevents NFs from transforming into CAFs 24. Becker et al. reported that the promoters of the genes of glycolytic rate-limiting enzymes pyruvate kinase and lactic dehydrogenase (LDHA), in breast cancer-associated fibroblasts were hypermethylated, which increased the expression of pyruvate kinase M2 (PKM2) and LDHA 25. A similar study by Shan et al. provided further evidence to support the reverse “Warburg Effect” hypothesis by reporting that the pancreas-associated fibroblasts expressed elevated levels of glycolytic enzymes LDHA and PKM2, as well as, monocarboxylate transporter 4 (MCT4) responsible for lactate secretion 26. Moreover, activation of TGF-β signaling in CAFs increase oxidative stress, autophagy/mitophagy, and aerobic glycolysis, which may be associated with downregulation of caveolin-1 (Cav-1) 27-29. Integrin beta 4 (ITGB4)-overexpression in triple-negative breast cancer (TNBC) cells was found to provide the ITGB4 protein to the CAFs via exosomes, which induced BNIP3L-dependent mitophagy in CAFs, thereby increasing their glycolysis levels 30. Yan et al. deciphered another mechanism through which breast cancer cells-secreted exosomal miR-105 activated MYC signaling in CAFs to induce increased catabolism of glucose and glutamine 31.

It was observed that CAFs proliferated at an unexpectedly slower rate compared to matched NFs, which was in contrast to the proliferation rate of cancer cells, suggesting that increased glycolysis in CAFs did not efficiently increase the proliferation and growth of the CAFs. Bonuccelli et al. found that Cav-1-deficient stromal fibroblasts were sufficient to promote human tumor xenograft growth and angiogenesis. Proteomic analysis of Cav-1-deficient CAFs indicated the upregulation of glycolytic enzymes, such as PKM2 and LDH-B. The researchers speculated that Cav-1-deficient CAFs might contribute to tumor growth and angiogenesis by providing energy-rich metabolites (e.g., L-lactate) in a paracrine fashion 32. Sun et al. reported that hypoxia-induced oxidative ataxia-telangiectasia mutated protein kinase (ATM) promoted glycolytic activity in breast cancer-associated fibroblasts by phosphorylating glucose transporter 1 (GLUT1) at position S490 and increasing the expression of PKM2. *In vitro* and *in vivo* (orthotopic xenograft) results showed that hypoxic CAF-derived lactate, which provides metabolic coupling between CAFs and breast cancer cells, promoted breast cancer cell invasion by activating the TGF-β1/p38 MAPK/MMP2/9 signaling axis and fueling the mitochondrial activity in cancer cells 33. Another study demonstrated that when pancreatic cancer cells were exposed to a CAF-conditioned medium (CM), cancer cells exhibited enhanced oxidative phosphorylation, causing the mitochondria to enlarge significantly.

The pancreatic cancer cells expressed significantly higher levels of MCT1, fumarate hydratase (FH), and succinate dehydrogenase (SDH) 26. The overexpression of these enzymes further hints at a metabolic coupling between CAFs and cancer cells. Zhang and colleagues observed that CAFs-derived from oral squamous carcinoma (OSCC) exhibited significantly higher ITGB2 expression. Higher ITGB2 expression in CAFs was correlated with poor clinical characteristics and outcomes in OSCC patients, indicating that ITGB2 ^hi^ CAFs might promote OSCC cell proliferation 34. Moreover, a co-culture assay and heterotopic oral cancer model demonstrated that ITGB2-mediated lactate release in CAFs promoted OSCC cell proliferation. Mechanistically, ITGB2 enhances the glycolytic activity in CAFs through the regulation of the PI3K/AKT/mTOR pathway. The lactate produced by ITGB2 ^hi^ CAFs is taken up by tumor cells, where it is metabolized to produce NADH, which is then oxidized within the mitochondrial oxidative phosphorylation system (OXPHOS) to generate ATP. Fischi et al. observed that human prostate cancer (PCa) cells induced a high expression of GLUT1 and MCT4 in CAFs, which increased the glucose uptake and lactate output by the CAFs. In contrast, PCa cells, upon contact with CAFs, were reprogrammed for aerobic metabolism, which then exhibited decreased GLUT1 expression and increased lactate uptake via the lactate transporter MCT1 35. Therefore, it may be inferred that PCa cells do not rely on glucose for growth but rather utilize the lactate produced by CAFs to support their tricarboxylic acid (TCA) cycle, anabolic processes, and cell proliferation. The inhibition of MCT1 in tumor cells probably blocks the lactate shuttle effect, and thus, inhibits tumor growth. A similar phenomenon was observed in breast cancer-and bladder cancer-associated fibroblasts 36, 37, where increased lactate production was associated with the upregulation of MCT1 and MCT4. The lactate shuttle phenomenon has also been observed in nasopharyngeal carcinoma, where the researchers noticed that inhibiting MCT4 expression in activated CAFs significantly decreased the proliferation, invasion, and colony formation of nasopharyngeal carcinoma cells 38. Another study reported that the patients who exhibited a high MCT4 expression in TNBC mesenchymal cells presented a worse prognosis 39. The above studies demonstrated the significance of glycolysis in CAFs for tumor growth and also elucidated the phenomenon of lactate shuttle between CAFs and tumor cells.

### Amino acid metabolism

Amino acids are essential for tumor growth and development, and several studies have demonstrated that CAFs synthesize the amino acids required for tumor cell growth through the TCA cycle 40. Glutamine (Gln), an important source of carbon and nitrogen, is reported to play a key role in tumor anabolism, and thus, reducing glutamine levels in the TME affects the viability of tumor cells 41-43. Several studies have reported the crucial roles of Gln metabolism in the interaction between CAFs and tumor cells (Table 1). In ovarian cancer, CAFs were shown to generate high levels of Gln by glutamine synthetase (GS). The CAF-derived Gln was exported to the ovarian cancer cells and converted to glutamate by the enzyme glutaminase. This further supported tumor cell growth by anaplerosis (replenishment process of metabolic pathway intermediates) of the TCA 44. Co-targeting GS in CAFs and glutaminase in cancer cells disrupted CAF/tumor cell metabolic crosstalk, inducing tumor regression in an ovarian carcinoma mouse model 44. Additionally, in a recent study, it was found that glutamine dependence drove CAF migration from the glutamine-depleted core of tumors to more glutamine-rich areas. Glutamine deprivation promoted CAF migration and invasion, which in turn facilitated the movement of tumor cells toward nutrient-rich territories 45. CAF migration toward Gln was modulated by a polarized protein kinase B (AKT2), and depletion of the polarized AKT2 prevented the invasion of Gln-driven CAFs and the escape of the tumor cells from the original tumor site 45.

Besides synthesizing glutamine directly to provide nutrition to tumor cells, CAFs are also capable of producing other amino acids as nitrogen sources. Linares et al. observed that p62 ^k^° CAFs can resist glutamine deficiency by directly controlling the stability of activating transcription factor 4 (ATF4) through its p62-mediated polyubiquitination, and thus, maintain PCa cell proliferation 46. The expression of asparagine synthase (ASNS) and pyruvate carboxylase (PC) increases in the p62 ^k^° CAFs. ASNS moderates the synthesis of asparagine from aspartate, while PC mediates the production of oxaloacetate from pyruvate, the route through, which pyruvate enters the TCA cycle. The increased expression of ASNS and PC suggests that the TCA cycle is enhanced, with an increase in the levels of aspartic acid and asparagine. The p62-deficient CAFs produce aspartate and asparagine, which serve as nitrogen sources for the growth of PCa cells, thereby maintaining the survival of PCa cells in a glutamine-deficient environment 46.

Research suggests that crosstalk exists between human skin squamous carcinoma (HSSC) cells and CAFs in terms of amino acid metabolism 40. Aspartate and glutamate are exchanged between HSSC cells and CAFs via the aspartate/glutamate transporter, known as the solute carrier family 1 membrane 3 (SLC1A3) channel. When glutamate from cancer cells enters CAFs one of two things might occur. Either glutamate is generated through the TCA cycle to produce aspartate, which is subsequently utilized by CAFs to biosynthesize and maintain cell proliferation, or glutamate forms glutathione (GSH) in CAFs to balance the redox status of cells and promote ECM remodeling 40.

These findings demonstrated that CAFs form amino acid metabolic couplings with tumor cells, which enable them to cope with nutrient deprivation in the TME, subsequently allowing the tumors to utilize amino acids efficiently and rapidly to promote tumor growth and development. However, there are many amino acids involved in the metabolic crosstalk between CAFs and tumor cells that have not been determined yet. Further studies are required to decipher the metabolic functions of other amino acids in tumor-CAF interactions.

### Lipid metabolism

Fatty acids (FAs) are the main building blocks of lipids and can be funneled into various metabolic pathways to synthesize more complex lipid species. FAs contribute to the vast structural diversity of the cellular lipid pool, which in turn serves to regulate several biochemical processes in normal cells 47. In recent years, the importance of altered FA metabolism in cancer has received renewed interest since they, aside from having a principal role as structural components of the membrane matrix, are important secondary messengers, and can also serve as fuel sources for energy production 48, 49. However, few studies have investigated how dysregulated lipid metabolism in CAFs affects tumorigenesis. In pancreatic ductal adenocarcinoma (PDAC), stroma-associated pancreatic stellate cells (PSCs) undergo a shift in lipid metabolism and intracellular liposome remodeling during activation 50. PSCs, while being activated, lose neutral lipids, and the intracellular levels of lysophospholipids increase dramatically. Additionally, lysophosphatidylcholines (LPCs) are secreted into the TME in large quantities, a few of which are directly absorbed and utilized by PDAC cells for membrane lipid formation, while the remaining LPCs are hydrolyzed by autotaxin (ATX) secreted by PDAC cells to generate lysophosphatidic acid (LPA), which then activates the AKT2 pathway in PDAC cells via the LPA receptor (LPAR) 50. In a similar study, LPA from ovarian cancer cells was shown to induce a CAF glycolytic phenotype in peritumoral fibroblasts by upregulating the hypoxia-inducible factor (HIF-1α) levels via LPAR, indicating again that the crosstalk between CAFs and cancer cells is mutualistic 51. The ATX/LPA/LPAR signaling pathway affects tumor growth, proliferation, and metabolism, and is a potential therapeutic target for several tumors, including liver and non-small cell lung cancers (reviewed in 52). Finally, in a study on the migration of colorectal cancer (CRC), Gong et al. demonstrated that CAFs undergo a reprogramming of liposome metabolism 53, which involves an increase in the expression of fatty acid synthase (FASN) in CAFs. This results in the production of more FAs that are then taken up by CRC cells; the FAs then promote the migration of the CRC cells.

## The role of pro-glycolytic CAFs in the metabolic reprogramming of tumor cells

**Figure 2:**
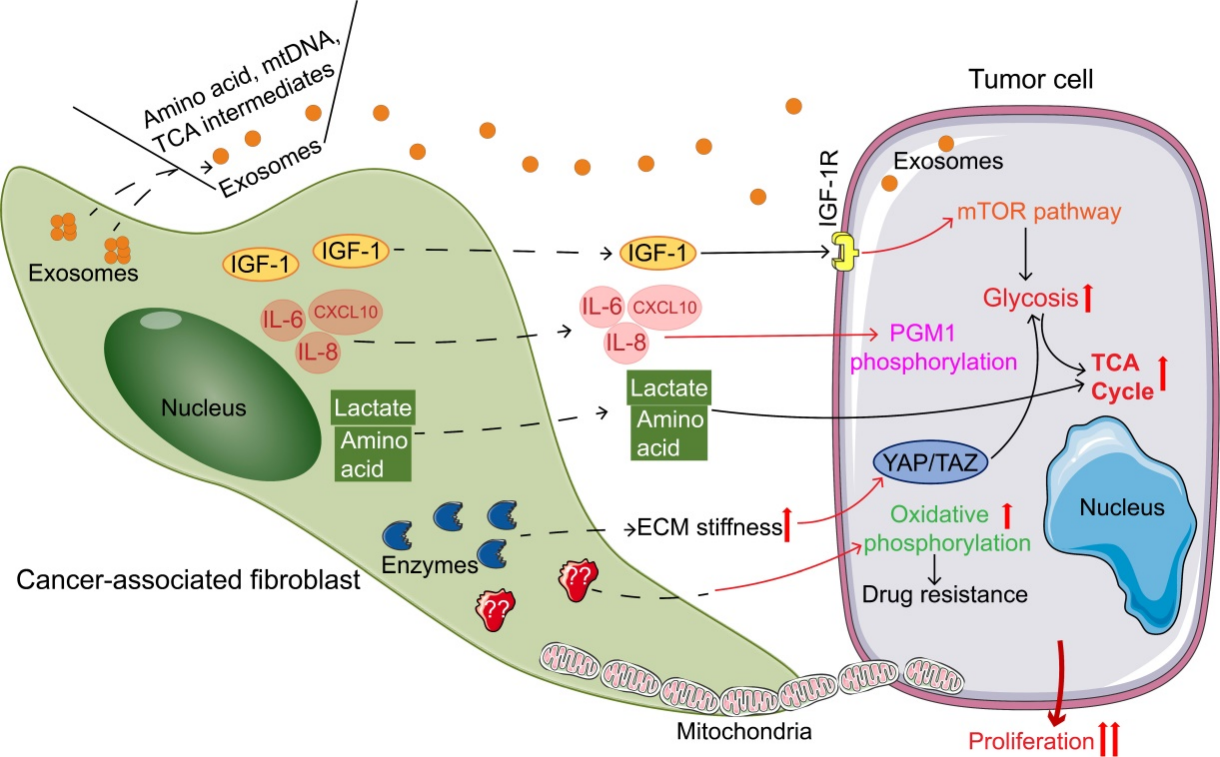
Cancer-associated fibroblasts (CAFs) regulating the metabolic reprogramming of tumor cells promote tumor growth. Pathways of CAFs regulating tumor metabolism: secreting exosomes containing nutrient; Secreting cytokines; directly secreting of amino acids and lactic acid; increasing ECM stiffness to regulate tumor metabolism; providing mitochondria; increasing oxidative metabolism. Abbreviations: IGF-1, insulin-like growth factor 1; IGF-1R, insulin-like growth factor 1 receptor; IL-6, interleukin-6; IL-8, interleukin-8; CXCL10, CXC chemokine ligand 10; mTOR, mammalian target of rapamycin; TCA, tricarboxylic acid; PGM1, phosphoglucomutase 1; ECM, extracellular matrix; YAP, Yes-related protein; TAZ, transcriptional co-activator PDZ binding motif.

In addition to undergoing metabolic reprogramming themselves, CAFs also play an important role in the metabolic reprogramming of tumor cells. CAFs regulate the metabolic remodeling of tumor cells by (I) directly/indirectly exporting nutrients, (II) providing mitochondria, (III) regulating the activity and oxidative properties of metabolic enzymes, and (IV) participating in ECM formation (Figure 2, Table 2).

### Directly/indirectly exporting nutrients

Several studies have confirmed that CAFs generate nutrients through catabolic processes and that these nutrients are then absorbed and utilized by tumor cells to maintain tumor growth. Sousa et al. reported that stroma-associated PSCs secrete alanine through activation of autophagy 54. Alanine outcompetes glucose and glutamine-derived carbon in PDAC to fuel the TCA cycle, and thus, the biosynthesis of non-essential amino acids and lipids. This further allows PDAC cells to divert carbon atoms from glucose to serine/glycine metabolism for sustaining their proliferation. The dysregulation of tryptophan metabolism due to the tumor-CAF interaction has also been demonstrated (Table 1) 54. Mishra et al. identified a Ras inhibitor, RASAL3, epigenetically silenced in the human prostatic CAF. Silencing of RASAL3 leads to oncogenic Ras activity that drives macropinocytosis-mediated glutamine synthesis 55. The glutamine generated by the CAFs is taken up by PCa cells, which increases the flux of the TCA cycle, enhances mitochondrial function and ATP production, and promotes tumor growth in PCa cells. In an orthotopic xenograft model, subsequent inhibition of macropinocytosis and glutamine transport had antitumor effects 55.

Exosomes are membrane-enclosed vesicles derived from the endosomal system during the formation of multivesicular bodies, with a diameter of ∼30-100 nm 56. In carcinogenesis, exosomes participate in proliferation, angiogenesis, immunosuppression, and preparation of pre-metastatic niches in secondary organs 57. Exosomes are rich in proteins, mRNA, miRNA, lipids, and other biologically active components, which exert certain effects by mediating the exchange of substances between the cells and altering the biological properties of recipient cells 58, 59. Recently, it has been reported that exosomes play an important role in the crosstalk between CAFs and cancer cells. Specific exosomes released from CAFs can be internalized by cancer cells and contribute to the progression and metastasis by transferring various types of substances (e.g., miRNA, proteins, mRNA, and lncRNAs) (reviewed in 60). Here, we focused on the delivery of metabolites from CAFs to tumor cells via exosomes.

Zhao et al. found that prostate CAF-derived exosomes could inhibit mitochondrial oxidative phosphorylation, thereby increasing glutamine-dependent reductive carboxylation and glycolysis in cancer cells. CAF-CM was collected to isolate exosomes. First, the samples were centrifuged at 2,000 g for 30 min to remove cells and debris; then, they were centrifuged at 10,000g for 60 min at 4 °C. The ^13^C isotope-labeling experiments had shown that exosomes could provide amino acids in nutrition-deficient cancer cells through a mechanism similar to macropinocytosis. The researchers performed Gas Chromatography-Mass Spectrometer (GC-MS) and ultrahigh-performance liquid chromatography (UPLC) experiments and confirmed that exosomes in both prostate and pancreatic CAFs contain complete metabolites, including amino acids, lipids, and TCA cycle intermediates, which are extensively used by cancer cells for carbon metabolism during nutritional deficiency or nutritional stress; thus, exosomes promote tumor growth 61, 62. Coincidentally, Kunou et al. investigated the roles of CAFs and their exosomes in the survival and drug resistance of lymphoma cells 63. CAFs were established from primary lymphoma samples, and exosomes secreted from CAFs were obtained by standard procedures, where the CAF-CM was initially collected and centrifuged at 300g for 10 min; then the supernatant was centrifuged at 2,000 g for 10 min. After filtering, the CM was finally ultracentrifuged at 32,000-35,000 rpm for 70 min. Glycolysis and ATP production in lymphoma cells increased in the presence of exosomes from CAFs. Metabolomic analysis indicated that the glucose 6-phosphate/ribose 5-phosphate ratio (G6P/R6P ratio), a parameter of glycolytic activity, was higher in tumor cells in the presence of exosomes from CAFs or in those cells co-cultured with CAFs, demonstrating increased glycolysis. Collectively, these data indicated that exosomes secreted from CAFs were, at least partly, involved in the improved survival of the lymphoma cells via increased glycolysis. Unfortunately, the study did not test whether the exosomes contained metabolites, and which components of the exosomes were associated with increased glycolysis in tumor cells. Altogether, the findings suggested that preventing exosomes from smuggling resources to starving cancer cells might be an effective strategy to treat cancers.

Besides directly delivering nutrients, exosomes derived from CAFs can upregulate glycolytic and glutamate-dependent reductive carboxylation capacity in cancer cells through the transfer of noncoding RNAs and substrates. Li et al. demonstrated that breast cancer cells reprogram the metabolic pathways after taking the exosomes secreted from CAFs 64. In this study, breast cancer patient-derived CAFs-secreted exosomes were isolated from CAF-CM. The CAF-CM was collected and centrifuged at 400g for 5 min. Next, cell debris was further removed from the supernatant by centrifugation at 3,000g for 20 min. After filtering the supernatant, it was ultracentrifuged at 110,000g for 4 h and the exosomes were collected. CAF-secreted exosomal lncRNA SNHG3 serves as a molecular sponge for miR-330-5p in breast cancer cells. Moreover, results of *in vitro* studies have shown that PKM can be targeted by miR-330-5p and is controlled by SNHG3 in breast cancer cells. Mechanistically, SNHG3 knockdown in CAF-secreted exosomes suppresses glycolysis metabolism and cell proliferation by the increase in miR-330-5p and decrease in PKM expression in tumor cells. SNHG3 functions as a miR-330-5p sponge to upregulate PKM expression, inhibit mitochondrial oxidative phosphorylation, increase glycolysis carboxylation, and enhance breast tumor cell proliferation 64. These observations suggest that the metabolic crosstalk between non-coding RNA-regulated CAFs and cancer cells might influence cancer progression and that improving the understanding of these processes may provide new directions for cancer treatment.

### Providing mitochondria

Altered mitochondrial function is reported to significantly regulate tumor metabolism 65. Surprisingly, CAFs may transfer their mitochondria to tumor cells. Zhang et al. demonstrated the export of mitochondria from CAFs to OSCC cells, both *in vivo* and *in vitro* 66. Ippolito et al. observed a unidirectional transfer of mitochondria from CAFs to PCa cells via cytoplasmic bridges 67. However, none of these studies elaborated on the functions and effects of the CAF-derived mitochondria within the tumor cells; thus, a great deal of research regarding this remains to be conducted.

Interestingly, the CAF-derived exosomes also provide the mitochondrial genome to the tumor cells. In drug-resistant breast cancer, the intact mitochondrial genome (mitochondrial DNA, mtDNA) of CAFs was observed to be packaged in the exosomes and transferred to breast cancer cells. The CAF-derived mtDNA can be expressed in an intact form within the breast cancer cells and can increase the levels of tumor oxidative phosphorylation and mitochondrial metabolism, which further enables tumor drug resistance and enhances the self-renewal potential of tumor stem cells 68.

### Regulating the activity and oxidative properties of metabolic enzymes

In 2015, our research team had observed that thymic fibroblast specific protein-1 (FSP-1)-positive fibroblasts released high amounts of IL-6, fibroblast growth factor 7 (FGF7), and S100A4 in the culture medium, which are crucial for the maintenance and regeneration of thymic epithelial cells 69. Ovarian cancer cells were also reported to induce their CAFs to secrete cytokines, such as IL-6, IL-8, and CXCL10 via TGF-β. These cytokines then induced the phosphorylation of phosphoglucomutase 1 (PGM1), which promoted glycogen catabolism and activated glycolysis and the pentose phosphate pathway (PPP) in ovarian cancer cells, leading to the proliferation, invasion, and early metastasis of cancer cells 70. Zhao et al. found that the pro-metastatic properties of CAFs *in vitro* and* in vivo* were associated with an elevated expression of the chemokine CXCL14. CXCL14-high CAFs mediated upregulation of LINC00092 in ovarian cancer cells, the levels of which also correlated with poor prognosis in patients. Mechanistic studies have shown that LINC00092 binds to a glycolytic enzyme, the fructose-2,6-biphosphatase PFKFB2, thereby promoting metastasis by altering glycolysis in cancer cells and sustaining the local supportive function of CAFs 71. In a study on the head and neck squamous cell carcinomas (HNSCC), CAFs were observed to secrete large amounts of hepatocyte growth factor (HGF) under the influence of lactate produced by HNSCC cells 72. The HGF secreted by CAFs subsequently induced the upregulation of the key enzymes of glycolysis (hexokinase II and phosphofructokinase) in HNSCC cells, thus promoting glycolysis in cancer cells. The results from a recent study showed that depletion of focal adhesion kinase (FAK) in a subpopulation of CAFs regulates paracrine signals that increase malignant cell glycolysis and tumor growth. Mechanistically, FAK depletion in CAFs increases chemokine Ccl6 and Ccl12 production which activates protein kinase A in cancer cells via CCR1/CCR2, leading to enhanced glycolysis in malignant cells 73. These results aid in establishing the mechanism of CAF-regulated and chemokine-mediated control of cancer metabolism and identifying the potential novel targets for anticancer therapy.

Besides affecting the key enzymes of metabolic pathways, CAFs can also influence the metabolism-related signaling pathways in tumor cells. Tommelein et al. reported that preoperative radiotherapy (RT) for CRC elicits secretion of insulin-like growth factor 1 (IGF-1) from CAFs 74. The binding of IGF-1 to the IGF-1 receptor on the CRC cells activates the mammalian target of the rapamycin (mTOR) pathway in the CRC cells, resulting in glucose uptake and lactate release. This increases the expression of the CRC cell channel protein SLC7A11 (solute carrier family 7, membrane 11), and promotes glutamine uptake by the CRC cells, thereby enhancing the resistance of CRC to RT treatment.

The redox state of tumors is often imbalanced, as shown by the increased levels of reactive oxygen species (ROS), which are important for both drug resistance and immune tolerance. Broekgaarden et al. reported that in an orthotopic xenograft model of PDAC, the tumors co-implanted with PDAC cells and CAFs exhibited an increased oxidative state and significantly increased resistance to oxaliplatin, compared to the tumors injected only with PDAC cells; the half-maximal inhibitory concentration (IC50) increased by 2.4-fold 75. Chan et al. observed that higher H_2_O_2_ production by CAFs was associated with impaired TGFβ signaling that led to the suppression of the antioxidant enzyme glutathione peroxidase 1 (GPX1). This consequently increased ROS levels and fueled tumor growth 76. On the contrary, it has been reported that CAFs can enhance platinum-based drug resistance in PCa cells by inhibiting drug accumulation and counteracting drug-induced oxidative stress, which is associated with increased glutathione levels in cancer cells 77. Thus, as a key regulator of tumor-CAF metabolic interactions, ROS can strongly influence the behavior and function of CAFs and tumor cells.

### Participating in the formation of the ECM

Tumor tissues contain a large number of ECM molecules, which besides maintaining the mechanical tension of the matrix, also communicate with cells via cell membrane surface receptor integrins 78. Interestingly, the adhesion of cells to the ECM also plays an important role in cell metabolism. When the cells detach from the ECM, glucose metabolism and mitochondrial respiration are inhibited, resulting in reduced cellular energy synthesis and increased intracellular ROS 79. In tumorigenesis, CAFs mediate the formation of ECM by secreting the enzymes associated with stromal remodeling 14, 80, 81. In squamous cell carcinoma, CAFs were observed to increase the stiffness of ECM, which activated the Yes-related protein (YAP)/transcriptional co-activator PDZ binding motif pathway and induced the expression of YAP downstream genes, glutaminase (GLS1), LDHA, and SLC1A3, thereby activating the glycolytic and glutamine metabolic pathways within the tumor cells. Additionally, the enhanced stiffness of the ECM induced amino acid exchange between cancer cells and their CAFs, thus promoting tumor proliferation 40. Similarly, Liu et al. also demonstrated that stiffer ECM-induced YAP activation depends on the JNK and p38 MAPK signaling cascades. YAP activation promotes hepatocellular carcinoma (HCC) cell migration depending on their accelerated aerobic glycolysis 82. These data indicate that ECM remodeling is directly influenced by CAFs, and ECM remodeling, in turn, influences CAFs in cancer. Moreover, these changes in the ECM directly or indirectly influence the metabolic phenotype and progression of tumors.

## Effect of CAFs on Systemic Metabolism in cancer

Besides causing metabolic dysregulation of local tissues, cancer is also associated with altered metabolism in the host 83. Cachexia is one of the main manifestations of the abnormal systemic metabolic responses to cancer, which affects more than 80% of cancer patients 84. Cancer cachexia is characterized by skeletal muscle protein loss and reduction of body lipid stores during metabolic processes 85. Cachexia has been mechanistically linked to the inflammatory response in cancer. Several pro-inflammatory cytokines, like IL-1, IL-6, and TNF-α, may play crucial roles in the pathological mechanisms of cancer cachexia 85, 86. Although the mechanisms driving cancer cachexia are still unclear, early evidence suggests that CAFs may play a role in tissue wasting, either by mediating inflammatory responses or through direct interaction with host tissues. It is well-known that fibroblast activation protein-α (FAP-α) marks reactive fibroblasts in the tumor stroma and the healing dermal scars 87, as well as, in chronic inflammatory lesions, such as primary biliary cirrhosis 88, atherosclerosis 89, and rheumatoid arthritis 90. FAP-α-positive CAFs in the primary liver tumor microenvironment have been verified to be associated with immunosuppression, enhancing recruitment of myeloid-derived suppressor cells (MDSC) by secreting Ccl2 91. However, it was recently found that FAP-α-positive fibroblasts were present in most tissues of transgenic mice permitting a FAP reporter 92. These FAP-α-positive fibroblasts were mainly derived from skeletal muscle, adipose tissue, and pancreas, and had highly similar transcriptomes, suggesting a common lineage. Experimental ablation of these cells in healthy mice caused an atrophic muscle response, just like a cachexia-like syndrome, characterized by rapid weight loss and reduced muscle mass despite adequate food intake. FAP-α-positive cells in the skeletal muscle are the major local sources of follistatin, key regulators of myofiber thickness and muscle growth. Surprisingly, a loss of FAP-α-positive cells in the skeletal muscle was found in cachectic tumor models, suggesting a causal role of the FAP-α-positive fibroblasts in the wasting of muscles in cachexia 92. These findings imply that fibroblasts are involved in the maintenance of muscle mass, and the loss of fibroblast from skeletal muscle can promote muscle wasting in cancer cachexia, thus significantly affecting systemic metabolism.

Increased systemic levels of pro-inflammatory cytokines are considered to be strongly linked to host metabolic disturbances associated with cancer progression 85. Many studies have focused on the cytokine IL-6 as a mediator of wasting syndromes associated with cachexia 93-96. It is well-established that CAFs are the main source of IL-6 in TME in different types of tumors 97-99, indicating a potential link between CAFs and cancer cachexia through the production of IL-6. In pre-cachectic mice with transplanted CRC or autochthonous PDAC, Thomas et al. found that IL-6 reduces the hepatic ketogenic potential through suppression of peroxisome proliferator-activated receptor alpha (PPARα), the transcriptional master regulator of ketogenesis 95. These mice, suffering from hypoketonemia, demonstrated a marked rise in glucocorticoid levels in response to caloric restriction. Interestingly, tumor-induced IL-6 impaired the ketogenic response, which increased systemic glucocorticoids that blocked anticancer immunotherapy 95. These findings support the possibility that, besides their involvement in local anti-tumoral immunity, CAFs may participate in a complex metabolic and inflammatory host response, which in turn may lead to systemic elevation of glucocorticoids and immunosuppression. But the role of IL-6 specifically derived from CAFs in this process remains to be further elucidated.

Besides focusing on the tumor-supporting role of CAFs, we also need to pay attention to some “alternative” studies. Three published papers simultaneously mentioned the protective effect of CAFs against PDAC, which may be related to the inhibition of pro-cachectic mechanisms 100-102. After the investigators removed Shh-dependent CAFs or α-SMA-positive CAFs using genetic or pharmacological approaches, they consistently found that CAF ablation accelerated tumor progression instead of inhibiting the process. Importantly, in a study, Rhim et al. noted that after removal of Shh-dependent CAFs, mice develop very small tumors with severe cachexia, along with the wasting of adipose tissue and muscle 101. These findings led us to consider the possibility that pancreatic CAFs might improve survival partly by inhibiting pro-cachectic mechanisms within the primary tumor. Unfortunately, however, a mechanistic link between Shh-dependent CAF function and key mediators of cancer cachexia has not yet been determined. The determination of the molecular mechanisms alleviating cachexia is of great significance to achieve optimal results in the treatment of PDAC. Therefore, further studies on the Shh-CAF axis are necessary.

## Concluding Remarks and Therapeutic Implications

Tumor cells must be capable of thriving in a nutrient-deprived tumor microenvironment to survive, a process in which CAFs assist the tumors to cope with the nutrient deprivation and maintain tumor growth, metastasis, and drug resistance 103. On the one hand, CAFs undergo metabolic reprogramming in the tumor microenvironment, which increases aerobic glycolysis in CAFs, producing a large number of metabolites that serve as sources of nutrients and energy for tumors to maintain tumor biosynthesis. On the other hand, tumor cells undergo metabolic reprogramming in response to the nutritional stress exerted by the tumor microenvironment; CAFs participate in and contribute to tumor metabolic reprogramming in various ways. The mechanism of an oxygen-independent metabolic switch from oxidative phosphorylation to aerobic glycolysis in CAFs has not been determined yet. Hypoxia has been implicated in the metabolic reprogramming of cancer cells, and HIF-1α has been shown to play an important role in the regulation of glycolysis 35, 104. Zhang et al. proposed that a decrease in IDH3α reduces the ratio of α-ketoglutarate (α-KG) to succinate and fumarate, resulting in a stabilization of HIF-1α under normoxic conditions, which, in turn, promotes glycolysis in CAFs 24. Likewise, Becker et al. identified the chronic hypoxia-induced epigenetic rewiring of fibroblasts that promotes a proglycolytic phenotype and results in the sustained elevation of HIF-1α, PKM, and LDHA and the suppression of fructose-bisphosphatase 1 (FBP1) gene expression 25. They found that chronic exposure to hypoxia in human NFs was sufficient to induce a transcriptome characteristic of CAFs (pro-glycolytic) despite re-oxygenation, suggesting hyperresponsiveness to hypoxia. The catalytic domain of Tet hydroxylases is dependent on the TCA cycle intermediate α-KG 105. A connection between oxygen tension and DNA methylation was shown, which possibly indicated changes in TCA cycle intermediates, one-carbon metabolism, and the activity of enzymes regulating DNA methylation. However, it remains unknown whether specific directives enable a DNA methylation pattern that supports a proglycolytic phenotype of CAFs or whether CAFs with newly acquired pro-glycolytic gene methylation patterns are selected due to microenvironmental pressure, such as oxygen availability. Although the study by Becker et al. implicated α-SMA-positive CAFs in breast cancer as tumor-promoting, Özdemir et al. reported that depletion of α-SMA-positive CAFs led cells in the non-invasive precursor stage or PDAC stage to become invasive, undifferentiated tumors with enhanced hypoxia, show an epithelial-to-mesenchymal transition and transform into cancer stem cells, thus adversely affecting animal survival 102. These results underscore the functional heterogeneity of CAFs and caution against assigning CAF markers with general functions across tumor types 15, 106.

An increasing number of studies are focusing on tumor metabolism, and within this area of research, the regulation of tumor metabolic reprogramming by CAFs is an important direction with a strong academic research value 107. Numerous studies have demonstrated that the presence of CAFs induces the metabolic reprogramming of tumors, promotes tumor adaptation to nutritional deprivation, and sustains tumorigenesis and progression 55. Moreover, *in vivo* studies have demonstrated that inhibiting the exchange of substances between CAFs and tumor cells, such as by the lactate shuttle 30 or the exchange of amino acids 44, may significantly inhibit tumor growth. Additionally, the metabolic reprogramming of CAFs leads to clinical tolerance of the therapeutic drugs in patients 108.

However, most of these studies are currently at a fundamental level, and there is a lack of reported clinical studies. Moreover, several obstacles need to be overcome, such as the limited number of methods available to measure the metabolism of CAFs, the uncertainty in metabolic heterogeneity, and the lack of metabolic similarity under *in vivo* and *in vitro* conditions. Therefore, it is imperative to understand the impact of tumor heterogeneity and the failure to recognize the nonlinear relationship between micronutrients and cancer in fundamental research and clinical trials.

## Figures and Tables

**Table 1:** Summary of the CAF-tumor cell crosstalk metabolic studies.

Cancer Type	CAF marker	Isolation protocols of CAFs	*In vitro* experiments	*In vivo* model	Findings (*In vitro* and *In vivo*)	Refs
BC	Cav-1	Cav-1 (-/-) deficient stromal fibroblasts derived from Cav-1 (-/-) deficient mice.	NA	Human tumor xenograft model: co-injection with human breast cancer cells and WT/Cav-1 (-/-) deficient stromal fibroblasts.	Cav-1-deficient stromal fibroblasts promoted both tumor growth and angiogenesis, and recruited Cav-1 (+) micro-vascular cells.	32
Possible mechanisms: Cav-1-deficient CAFs upregulate the expression of glycolytic enzymes, PKM2 and LDH-B. Cav-1-deficient CAFs may contribute toward tumor growth and angiogenesis, by providing energy-rich metabolites in a paracrine fashion.						
BC	FN; α-SMA; FAP	CAFs and NFs were isolated from breast tumor tissues and their paired normal tissues.	Engineered CAFs (sh-ATM, sh-GLUT1, sh-PKM2 and sh-MCT4) were established; Preparation of NFs-CM and CAFs-CM; Cell migration and invasion assay;	Orthotopic xenografts: subcutaneously co-injection with BC cells and control CAFs (CAF/Ctrl) or engineered CAFs (CAF/sh-ATM, CAF/sh-MCT4).	In vitro, BC cells showed a reduced migration or invasion ability after co-cultured with CM from CAFs/shATM; In vivo, the tumor burden mice, which co-injected with BC cells and engineered CAFs had a significant small tumor and fewer lung metastases.	33
Possible mechanisms: Hypoxia-induced oxidized ATM promotes glycolytic activity of CAFs by phosphorylating GLUT1 at S490 and increasing PKM2 expression. Lactate derived from hypoxic CAFs promotes BC cells invasion by activating the TGFβ1/p38 MAPK/MMP2/9 signaling.						
OSCC	α-SMA	CAFs and NFs were obtained from tumor samples and the matched adjacent non-tumor tissues of patients with OSCC.	Preparation of NFs-CM and CAFs-CM; Cell proliferation assay;	Xenografts: HSC3-Ctrl & CAFs-Ctrl, HSC3-Ctrl & CAFs-ITGB2, HSC3-shMCT1 & CAFs-Ctrl, HSC3-shMCT1 & CAFs-ITGB2 were subcutaneously co-injected into the flanks of NOD/SCID mice, respectively.	In vitro, OSCC cells showed a reduced growth rates and the colony forming number after co-cultured with CM from CAFs/shITGB2; In vivo, tumors of the CAFs-ITGB2 and HSC3-Ctrl group grew more quickly and were larger.	34
Possible mechanisms: ITGB2 regulates PI3K/AKT/mTOR pathways to enhance glycolysis activity in CAFs. Lactate derived from ITGB2-expressing CAFs is absorbed and metabolized in OSCC to generate NADH, which is then oxidized in the mitochondrial OXPHOS to produce ATP.						
PCa	α-SMA	Healthy human prostate fibroblasts and CAFs were isolated from patients with benign prostatic hyperplasia or aggressive PCa.	Preparation of NFs-CM and CAFs-CM; Cell proliferation assay;	Xenografts: Wild type or MCT1 silenced PCa were subcutaneously injected in SCID-bg/bg together with CAFs.	In vitro, PCa cells showed an increased proliferation index and the colony forming number after co-cultured with CM from CAFs; In vivo, co-injection of CAFs with PCa cells strongly enhanced the tumor growth rate.	35
Possible mechanisms: PCa cells, upon contact with CAFs, are reprogrammed with a decrease in GLUT1 expression and an increase in lactate upload via MCT1. PCa cells gradually become dependent of lactate to drive anabolic pathways and thereby cell growth.						
NPC	α-SMA; FAP; vimentin	The primary NFs and CAFs of NPC were isolated from 3 pairs of tumor tissues and adjacent normal tissues.	NPC cells were cocultured with NFs treated by NPC-CM-derived EVs; Cell proliferation assay; Cell migration (Transwell assay);	Xenografts: NPC cells were subcutaneously injected in each mouse together with NFs or NFs treated by NPC-CM-derived EVs.	In vitro, co-culture with CAFs promoted the proliferation, migration and radiation resistance of NPC cells; In vivo, EV packaged LMP1 promoted tumor proliferation and pre-metastatic niche formation by activating CAFs.	38
Possible mechanisms: NPC-CM-derived EVs packaged LMP1-activated CAFs upregulate MCT4 by activating the NF-κB p65 pathway to export lactate and β-HB into cancer cells expressing MCT1 to import mitochondrial fuel for OXPHOS.						
OVCA	Not mentioned	Ovarian CAFs were derived from advanced-stage high-grade serous OVCA samples and NFs were derived from normal ovaries obtained from patients with benign gynecologic malignancies.	Preparation of NFs-CM and CAFs-CM; Cell proliferation assay;	Orthotopic OVCA Mouse Model: surgical implantation of control siRNA, OVCA-GLS siRNA, CAFs-Glul siRNA, or combinations of GLS and Glul siRNA cells directly into the left ovary, respectively.	In vitro, CM derived from CAFs rescued cancer cells proliferation; In vivo, co-targeting Glul in CAFs and GLS in OVCA cells reduced tumor weight, nodules, and metastasis.	44
Possible mechanisms: CAFs boost glutamine production by harnessing carbon and nitrogen from atypical nutrient sources to maintain cancer cell growth when glutamine is scarce.						
PCa	FSP-1	Human prostate stromal fibroblasts (WPMY-1) in which p62 was inactivated either by CRISPR-mediated gene editing (sgp62), or by shRNA-mediated knockdown (shp62).	Preparation of prostate stromal fibroblasts-CM; Cell proliferation assay; Organotypic cultures using PCa cells and prostate stromal fibroblasts; Cell invasion assay;	NA	In vitro, p62-deficiency in the stroma can sustain PCa proliferation in the absence of Gln in human and mouse co-cultures. In vitro, p62^-/-^ fibroblasts enhanced the invasiveness and proliferation index of PCa cells.	46
Possible mechanisms: p62 deficiency in stromal fibroblasts promotes resistance to glutamine deprivation by the direct control of ATF4 stability through its p62-mediated polyubiquitination.						
PDAC	α-SMA	Primary CAF lines were isolated from disaggregated primary PDAC tumor tissue. Primary PSCs were isolated from mouse healthy pancreata.	Preparation of CAFs-CM and PSCs-CM; Cell proliferation assay; Cell invasion assay;	Xenografts: subcutaneous co-transplantation assays in immune-compromised mice with both PDAC cells and PSCs.	In vitro, stromal cell CM elicited a concentration-dependent increase in PDAC cells proliferation. PSCs CM promoted migration of PDAC cells. In vivo, PSCs increased both tumor growth and intratumoral LPA levels.	50
Possible mechanisms: PSC secreted lysophospatidylcholines promoted the secretion of oncogenic autotaxin-LPA, which supported proliferation, migration and AKT activation in PDAC.						
CRC	Vimentin; α-SMA	The fibroblasts isolated from CRC tissues were defined as CAFs and the fibroblasts from normal adjacent tissues as NFs.	Preparation of NFs-CM and CAFs-CM; Engineered CAFs (CAF siFASN) were established; Wound-healing assay; Transwell assay;	Xenografts: CRC cells were mixed or not mixed with an equal number of CAFs or NFs and subcutaneously injected into the spleen of 7-week-old female nude mice.	In vitro, the transwell assay and wound-healing assay showed CM from siFASN CAF reduced CRC cells migration. In vivo, the mice injected with the mixture of CRC cells and CAFs had more metastasis in liver and the loss of CD36 reduced the metastasis.	53
Possible mechanisms: FASN-dependent CAFs-secreted lipids were taken up by tumor cells and induced tumor migration capacity.						
PDAC	α-SMA; Desmin	Human PSCs were isolated from an untreated human PDAC resection; Primary CAFs were isolated from tumor resections; mouse PSCs were generated from B6 females harboring mouse PDAC.	Preparation of human PSCs-CM; Cell proliferation assay; Metabolism experiments	Xenografts: PDAC cells were either injected alone or co-injected with human PSCs/mouse PSCs previously infected with shGFP, shATG5 or shATG7 shRNAs into the flanks of nude female mice.	In vitro, human PSCs-CM increased PDAC OCR. When grown in a low-nutrient setting, there was a significant positive effect of PSCs-CM on PDAC proliferation. In vivo, both tumor take and tumor growth kinetics were increased significantly when co-injected with autophagy-competent PSCs, and this increase was significantly attenuated when PDAC cells were co-injected with autophagy-incompetent PSCs.	54
Possible mechanisms: Autophagy dependent-alanine secretion by PSCs became an alternative carbon source for cancer cells. This led to an increase in the OCR of PDAC cells.						
PCa	tenascin C; FAP; MMP1; MMP3	Prostatic fibroblasts were isolated from PCa patients and mouse prostates. These fibroblasts were xenografted with nontumorigenic BPH1 prostatic epithelia. The fibroblasts were termed CAFs only if tumors developed within 4 weeks of grafting; otherwise, the non-tumor inductive fibroblasts were termed NFs.	A 3D organotypic coculture system were established; Seahorse XF-24 metabolic flux analysis; Cell proliferation assay; Cell migration (Transwell assay);	Tissue recombination mouse models: Cell recombinants were prepared by mixing epithelial cells with CAFs or stromal cells expressing Ras^V12^ per site in collagen. Orthotopic grafting constituted the placing of the collagen plugs in the 2 anterior lobes of the prostate, and kidney grafts were placed under the renal capsule of C57BL/6 male 8-week-old mice. The mice were either left intact or castrated 1 week after grafting.	In vitro, PCa cells were significantly more proliferative when cultured with Ras^V12^-expressing fibroblasts. A significant proliferative induction of cancer cells when associated with Rasal3-KO fibroblasts. Metabolome analysis of PCa cells demonstrated glutamine and glutamate to be significantly elevated when exposed to CAFs-CM compared with NFs-CM.	55
Possible mechanisms: A Ras inhibitor, Rasal3 as epigenetically silenced in human prostatic CAF, leading to oncogenic Ras activity driving macropinocytosis-mediated glutamine synthesis, which potentiates growth of adjacent epithelial.						
OVCA	CXCL14	CAFs and NFs were isolated from same ovarian site in 10 EOC and 10 non-cancerous prophylactic oophorectomy specimens.	Preparation of CAFs siCXCL14-CM and CAFs control-CM; Wound healing analysis;	Xenografts: an orthotopic model generated by intrabursal injection of OVCA cells alone or together with either CAFs or CXCL14-silenced CAFs.	In vitro, OVCA cells incubated with CM from CAFs transfected with CXCL14-siRNAs demonstrated significantly decreased migratory capacity and increased anoikis rate. CXCL14-positive CAFs induce overexpression of LINC00092 in vitro and in vivo. In vivo, mice inoculated with OVCA cells mixed with CXCL14-silenced CAFs demonstrated significantly decreased peritoneal metastasis.	71
Possible mechanisms: LINC00092 is induced upon stimulation by CAF-secreted CXCL14 in OVCA. LINC00092 bound a glycolytic enzyme, the fructose-2,6-biphosphatase PFKFB2, thereby maintenance of metastatic and glycolytic phenotype of OVCA by altering glycolysis and sustaining the local supportive function of CAFs.						
BC; PDAC	FSP-1; PDGFR-β	CAFs were isolated from MMTV+; FSP-Cre-; FAK^fl/fl^ and MMTV+; FSP-Cre+; FAK^fl/fl^ mice.	Preparation of CAFs-CM; Seahorse XF96^e^ metabolic flux analysis;	FSP-Cre+; FAK^fl/fl^ and control FSP-Cre-; FAK^fl/fl^ mice were injected orthotopically with either syngeneic breast cancer cells or pancreatic ductal adenocarcinoma cells. FSP-Cre+; FAK^fl/fl^ and FSP-Cre-; FAK^fl/fl^ mice were also crossed with MMTV-PyMT mice to generate MMTV+; FSP-Cre+; FAK^fl/fl^ and MMTV+; FSP-Cre-; FAK^fl/fl^ mice that developed spontaneous breast tumors.	In vitro, CM from FAK-depleted CAFs enhanced the glycolysis, glycolytic capacity and glycolytic reserve of malignant cells significantly; In vivo, CAF FAK depletion increased breast and pancreatic cancer growth; FAK depletion in CAFs enhanced chemokine Ccl6 and Ccl12 production;	73
Possible mechanisms: FAK-deletion in CAFs induced malignant cell glycolysis and tumor growth via CCR1/CCR2.						

Abbreviations: CAF, cancer-associated fibroblast; BC, breast cancer; Cav-1, caveolin-1; NA, not application; WT, wild type; PKM2, pyruvate kinase M2; LDH-B, lactate dehydrogenase B; FN, fibronectin; α-SMA, α-smooth muscle actin; FAP, fibroblast activation protein; NF, normal fibroblast; ATM, ataxia-telangiectasia mutated protein kinase; GLUT1, glucose transporter 1; MCT4, monocarboxylate transporter4; CM, conditioned medium; Ctrl, control; TGFβ, transforming growth factor-beta; MAPK, mitogen-activated protein kinase; MMP, matrix metalloproteinase; OSCC, oral squamous cell carcinoma; ITGB2, integrin beta 2; PI3K, phosphoinositol 3 kinase; AKT, protein kinase B; mTOR, mammalian target of rapamycin; NADH, oxidative phosphorylation system; OXPHOS, oxidative phosphorylation system; ATP, triphosadenine; PCa, prostate cancer; NPC, nasopharyngeal carcinoma; EVs, extracellular vesicles; LMP1, latent membrane protein 1; β-HB, β-hydroxybutyrate; OVCA, ovarian cancer; GLS, glutaminase; Glul, Gln synthetase; FSP-1, fibroblast-specific protein-1; ATF4, activating transcription factor 4; PDAC, pancreatic ductal adenocarcinoma; PSCs, pancreatic stellate cells; LPA, lysophosphatidic acid; CRC, colorectal cancer; FASN, fatty acids synthase; ATG5, autophagy Related 5; OCR, oxygen consumption rate; CXCL14, C-X-C Motif Chemokine Ligand 14; PFKFB2, 6-phosphofructo-2-kinase/fructose-2,6-biphosphatase 2; PDGFRβ, platelet-derived growth factor receptor beta; FAK, focal adhesion kinase; Ccl6, Chemokine (C-C motif) ligand 6; CCR1, chemokine receptor 1.

**Table 2:** CAFs regulate the metabolic remodeling of tumor cells in several ways.

Type	Tumor	Possible mechanism	Ref.
Exporting nutrients			
Directly	PDAC	The autophagic PSCs cells stimulated by cancer cells exports alanine to provide energy for cancer cells	54
	PCa	Silencing RASAL3 expression contributes to the activation of the Ras pathway, which in turn stimulates CAFs to translocate albumin to lysosomes for degradation and release of glutamine via endocytosis	55
Indirectly	PCa	Prostate CAFs-derived exosomes can inhibit mitochondrial oxidative phosphorylation, thereby increasing glutamine-dependent reductive carboxylation and glycolysis in cancer cells	61
	lymphoma	Glycolysis and ATP production in lymphoma cells increase in the presence of exosomes from CAFs	63
	BC	Exosomal lncRNA SNHG3 secreted by CAFs decreases the level of miR-330-5p in cancer cells, which in turn increases the expression of PKM	64
Providing mitochondria			
	OSCC	Mitochondria exported from CAFs to cancer cells	66
	PCa	Unidirectional transfer of mitochondria from CAFs to cancer cells via cytoplasmic bridges	67
	BC	The intact mtDNA) of CAFs is packaged in exosomes and transferred into breast cancer cells, which increases tumor oxidative phosphorylation levels and mitochondrial metabolism levels	68
Regulating metabolic enzymatic activity			
	Ovarian cancer	Cancer cells induce their CAFs to secrete cytokines such as IL-6, IL-8 and CXCL10 through TGF-β, which induces PGM1 phosphorylation to promote glycogen catabolism, activates glycolysis and pentose phosphate pathway in cancer cells	70
	Ovarian cancer	Exosomal LINC00092 derived from CAFs promoted cancer cell metastasis by binding to PFKFB2, which maintains the function of CAFs and promotes glycolysis in cancer cells	71
	HNSCC	HGF secreted by CAFs induces upregulation of key enzymes of glycolysis (hexokinase II and phosphofructokinase) in cancer cells	72
	BC; PDAC	FAK-depletion in CAFs increases chemokine Ccl6 and Ccl12 production which via CCR1/CCR2 on cancer cells, activate protein kinase A, leading to enhanced malignant cell glycolysis	73
	CRC	IGF-1 secreted by CAFs binding to the IGF-1 receptor on cancer cells activated mTOR pathway, causing glucose uptake and lactate release, increasing SLC7A11 expression and promoted glutamine uptake by cancer cells.	74
Regulating oxidative properties			
	PDAC	The presence of CAFs increases the oxidative properties of tumors and makes them resistant to drugs, but the mechanism is unclear.	75
	SCC	Higher H_2_O_2_ production by CAFs is contingent on impaired TGFβ signaling leading to the suppression of GPX1, which consequently increasing ROS levels and fueling tumor growth	76
	PCa	CAFs enhance platinum-based drug resistance in PCa cells by inhibiting drug accumulation and counteracting drug-induced oxidative stress, which is associated with an increased glutathione level in cancer cells	77
Participating in ECM formation			
	SCC	CAFs increase the stiffness of ECM, which activate the YAP/TAZ pathway in cancer cells and induce the expression of YAP downstream genes GLS1, LDHA, and SLC1A3, thereby activating the glycolytic pathway and glutamine metabolic pathway in tumor cells; and enhance ECM stiffness induced amino acid exchange between cancer cells and their CAFs, promoting tumor proliferation	40
	HCC	Stiffer ECM-induced YAP activation is depending on JNK and p38 MAPK signaling cascades. YAP activation promotes cancer cell migration depending on their accelerated aerobic glycolysis	82

Abbreviations: CAF, cancer-associated fibroblast; PDAC, pancreatic ductal adenocarcinoma; PSC, pancreatic stellate cell; PCa, prostate cancer; RASAL3, Ras protein activator like 3; PFKFB2, 6-phosphofructose-2-kinase/fructose-2,6-phosphatase 2; PKM, pyruvate kinase M2; OSCC, oral squamous cell carcinoma; BC, breast cancer; mtDNA, mitochondrial DNA; IL, interleukin; CXCL10, chemokine 10; TGF-β, transforming growth factor-β; PGM1, phosphoglucomutase 1; HNSCC, head and neck squamous cell carcinomas; FAK, focal adhesion kinase; Ccl6, Chemokine (C-C motif) ligand 6; CCR1, chemokine receptor 1; CRC, colorectal cancer; HGF, hepatocyte growth factor; IGF-1, insulin-like growth factor 1; SLC7A11, solute carrier family 7 membrane 11; ECM, extracellular matrix; YAP, Yes-related protein; TAZ, transcriptional co-activator PDZ binding motif; GLS1, glutaminase; LDHA, lactic dehydrogenase; SLC1A3, solute carrier family 1 membrane 3; SCC, squamous cell carcinoma; GPX1, glutathione peroxidase 1; ROS, reactive oxygen species; HCC, hepatocellular carcinoma.

## Abbreviations

TME: tumor microenvironment
CAFs: cancer-associated fibroblasts
ECM: extracellular matrix
CXCL12: C-X-C motif chemokine ligand 12
TNF-α: tumor necrosis factor
IL: interleukins
TGF-β: transforming growth factor β
FGF: fibroblast growth factor
VEGF: vascular endothelial factor
EGF: endothelial growth factor
NFs: normal fibroblasts
IDH3α: isocitrate dehydrogenase 3 complex
LDHA: lactic dehydrogenase
PKM2: pyruvate kinase M2
MCT4: monocarboxylate transporter 4
Cav-1: caveolin-1
ITGB4: Integrin beta 4
TNBC: triple-negative breast cancer
ATM: ataxia-telangiectasia mutated protein kinase
GLUT1: glucose transporter 1
FH: fumarate hydratase
SDH: succinate dehydrogenase
OSCC: oral squamous carcinoma
OXPHOS: mitochondrial oxidative phosphorylation system
PCa: prostate cancer
TCA: tricarboxylic acid
Gln: Glutamine
GS: glutamine synthetase
AKT2: protein kinase B
ATF4: activating transcription factor 4
ASNS: asparagine synthase
PC: pyruvate carboxylase
HSSC: human skin squamous carcinoma
SLC1A3: solute carrier family 1 membrane 3
GSH: glutathione
FAs: Fatty acids
PDAC: pancreatic ductal adenocarcinoma
PSCs: pancreatic stellate cells
LPCs: lysophosphatidylcholines
ATX: autotaxin
LPA: lysophosphatidic acid
LPAR: LPA receptor
HIF-1α: hypoxia-inducible factor
CRC: colorectal cancer
FASN: fatty acids synthase
GC-MS: Gas Chromatography-Mass Spectrometer
UPLC: ultrahigh-performance liquid chromatography
G6P/R6P ratio: glucose 6-phosphate/ribose 5-phosphate ratio
mtDNA: mitochondrial DNA
FSP-1: fibroblast specific protein-1
FGF7: fibroblast growth factor 7
PGM1: phosphoglucomutase 1
PPP: pentose phosphate pathway
PFKFB2: 6-phosphofructo-2-kinase/fructose-2,6-biphosphatase 2
HNSCC: head and neck squamous cell carcinomas
HGF: hepatocyte growth factor
FAK: focal adhesion kinase
IGF-1: insulin-like growth factor 1
mTOR: mammalian target of rapamycin
SLC7A11: solute carrier family 7, membrane 11
RT: radiotherapy
ROS: reactive oxygen species
IC50: half maximal inhibitory concentration
GPX1: glutathione peroxidase 1
YAP: Yes-related protein
GLS1: glutaminase
HCC: hepatocellular carcinoma
FAP-α: fibroblast activation protein-α
MDSC: myeloid-derived suppressor cells
PPARα: peroxisome proliferator-activated receptor alpha
α-KG: α-ketogluterate
FBP1: fructose-bisphosphatase 1

## References

[1] Faubert B, Solmonson A, DeBerardinis RJ (2020). Metabolic reprogramming and cancer progression. Science.

[2] Zhang W, Bouchard G, Yu A, Shafiq M, Jamali M, Shrager JB (2018). GFPT2-Expressing Cancer-Associated Fibroblasts Mediate Metabolic Reprogramming in Human Lung Adenocarcinoma. Cancer Res.

[3] Vander Heiden MG, DeBerardinis RJ (2017). Understanding the Intersections between Metabolism and Cancer Biology. Cell.

[4] Sun L, Suo C, Li ST, Zhang H, Gao P (2018). Metabolic reprogramming for cancer cells and their microenvironment: Beyond the Warburg Effect. Biochim Biophys Acta Rev Cancer.

[5] Arcucci A, Ruocco MR, Granato G, Sacco AM, Montagnani S (2016). Cancer: An Oxidative Crosstalk between Solid Tumor Cells and Cancer Associated Fibroblasts. Biomed Res Int.

[6] von Ahrens D, Bhagat TD, Nagrath D, Maitra A, Verma A (2017). The role of stromal cancer-associated fibroblasts in pancreatic cancer. J Hematol Oncol.

[7] Fiori ME, Di Franco S, Villanova L, Bianca P, Stassi G, De Maria R (2019). Cancer-associated fibroblasts as abettors of tumor progression at the crossroads of EMT and therapy resistance. Mol Cancer.

[8] Shiga K, Hara M, Nagasaki T, Sato T, Takahashi H, Takeyama H (2015). Cancer-Associated Fibroblasts: Their Characteristics and Their Roles in Tumor Growth. Cancers (Basel).

[9] Xing F, Saidou J, Watabe K (2010). Cancer associated fibroblasts (CAFs) in tumor microenvironment. Front Biosci (Landmark Ed).

[10] Kalluri R (2016). The biology and function of fibroblasts in cancer. Nat Rev Cancer.

[11] Öhlund D, Handly-Santana A, Biffi G, Elyada E, Almeida AS, Ponz-Sarvise M (2017). Distinct populations of inflammatory fibroblasts and myofibroblasts in pancreatic cancer. J Exp Med.

[12] Erez N, Truitt M, Olson P, Arron ST, Hanahan D (2010). Cancer-Associated Fibroblasts Are Activated in Incipient Neoplasia to Orchestrate Tumor-Promoting Inflammation in an NF-kappaB-Dependent Manner. Cancer Cell.

[13] Kobayashi H, Enomoto A, Woods SL, Burt AD, Takahashi M, Worthley DL (2019). Cancer-associated fibroblasts in gastrointestinal cancer. Nat Rev Gastroenterol Hepatol.

[14] Chen X, Song E (2019). Turning foes to friends: targeting cancer-associated fibroblasts. Nat Rev Drug Discov.

[15] Sahai E, Astsaturov I, Cukierman E, DeNardo DG, Egeblad M, Evans RM (2020). A framework for advancing our understanding of cancer-associated fibroblasts. Nat Rev Cancer.

[16] Ishii G, Ochiai A, Neri S (2016). Phenotypic and functional heterogeneity of cancer-associated fibroblast within the tumor microenvironment. Adv Drug Deliv Rev.

[17] Comito G, Ippolito L, Chiarugi P, Cirri P (2020). Nutritional Exchanges Within Tumor Microenvironment: Impact for Cancer Aggressiveness. Front Oncol.

[18] Chiarugi P, Cirri P (2016). Metabolic exchanges within tumor microenvironment. Cancer Lett.

[19] Sanford-Crane H, Abrego J, Sherman MH (2019). Fibroblasts as Modulators of Local and Systemic Cancer Metabolism. Cancers (Basel).

[20] Karta J, Bossicard Y, Kotzamanis K, Dolznig H, Letellier E (2021). Mapping the Metabolic Networks of Tumor Cells and Cancer-Associated Fibroblasts. Cells.

[21] Santi A, Kugeratski FG, Zanivan S (2018). Cancer Associated Fibroblasts: The Architects of Stroma Remodeling. Proteomics.

[22] Liberti MV, Locasale JW (2016). The Warburg Effect: How Does it Benefit Cancer Cells?. Trends Biochem Sci.

[23] Martinez-Outschoorn UE, Lin Z, Trimmer C, Flomenberg N, Wang C, Pavlides S (2011). Cancer cells metabolically "fertilize" the tumor microenvironment with hydrogen peroxide, driving the Warburg effect: implications for PET imaging of human tumors. Cell Cycle.

[24] Zhang D, Wang Y, Shi Z, Liu J, Sun P, Hou X (2015). Metabolic reprogramming of cancer-associated fibroblasts by IDH3α downregulation. Cell Rep.

[25] Becker LM, O'Connell JT, Vo AP, Cain MP, Tampe D, Bizarro L (2020). Epigenetic Reprogramming of Cancer-Associated Fibroblasts Deregulates Glucose Metabolism and Facilitates Progression of Breast Cancer. Cell Rep.

[26] Shan T, Chen S, Chen X, Lin WR, Li W, Ma J (2017). Cancer-associated fibroblasts enhance pancreatic cancer cell invasion by remodeling the metabolic conversion mechanism. Oncol Rep.

[27] Fuyuhiro Y, Yashiro M, Noda S, Kashiwagi S, Matsuoka J, Doi Y (2011). Upregulation of cancer-associated myofibroblasts by TGF-β from scirrhous gastric carcinoma cells. Br J Cancer.

[28] Guido C, Whitaker-Menezes D, Capparelli C, Balliet R, Lin Z, Pestell RG (2012). Metabolic reprogramming of cancer-associated fibroblasts by TGF-β drives tumor growth: connecting TGF-β signaling with "Warburg-like" cancer metabolism and L-lactate production. Cell Cycle.

[29] Sotgia F, Martinez-Outschoorn UE, Howell A, Pestell RG, Pavlides S, Lisanti MP (2012). Caveolin-1 and cancer metabolism in the tumor microenvironment: markers, models, and mechanisms. Annu Rev Pathol.

[30] Sung JS, Kang CW, Kang S, Jang Y, Chae YC, Kim BG (2020). ITGB4-mediated metabolic reprogramming of cancer-associated fibroblasts. Oncogene.

[31] Yan W, Wu X, Zhou W, Fong MY, Cao M, Liu J (2018). Cancer-cell-secreted exosomal miR-105 promotes tumour growth through the MYC-dependent metabolic reprogramming of stromal cells. Nat Cell Biol.

[32] Bonuccelli G, Whitaker-Menezes D, Castello-Cros R, Pavlides S, Pestell RG, Fatatis A (2010). The reverse Warburg effect: glycolysis inhibitors prevent the tumor promoting effects of caveolin-1 deficient cancer associated fibroblasts. Cell Cycle.

[33] Sun K, Tang S, Hou Y, Xi L, Chen Y, Yin J (2019). Oxidized ATM-mediated glycolysis enhancement in breast cancer-associated fibroblasts contributes to tumor invasion through lactate as metabolic coupling. EBioMedicine.

[34] Zhang X, Dong Y, Zhao M, Ding L, Yang X, Jing Y (2020). ITGB2-mediated metabolic switch in CAFs promotes OSCC proliferation by oxidation of NADH in mitochondrial oxidative phosphorylation system. Theranostics.

[35] Fiaschi T, Marini A, Giannoni E, Taddei ML, Gandellini P, De Donatis A (2012). Reciprocal metabolic reprogramming through lactate shuttle coordinately influences tumor-stroma interplay. Cancer Res.

[36] Whitaker-Menezes D, Martinez-Outschoorn UE, Lin Z, Ertel A, Flomenberg N, Witkiewicz AK (2011). evidence for a stromal-epithelial "lactate shuttle" in human tumors: MCT4 is a marker of oxidative stress in cancer-associated fibroblasts. Cell Cycle.

[37] Shi H, Jiang H, Wang L, Cao Y, Liu P, Xu X (2015). Overexpression of monocarboxylate anion transporter 1 and 4 in T24-induced cancer-associated fibroblasts regulates the progression of bladder cancer cells in a 3D microfluidic device. Cell Cycle.

[38] Wu X, Zhou Z, Xu S, Liao C, Chen X, Li B (2020). Extracellular vesicle packaged LMP1-activated fibroblasts promote tumor progression via autophagy and stroma-tumor metabolism coupling. Cancer Lett.

[39] Witkiewicz AK, Whitaker-Menezes D, Dasgupta A, Philp NJ, Lin Z, Gandara R (2012). Using the "reverse Warburg effect" to identify high-risk breast cancer patients: stromal MCT4 predicts poor clinical outcome in triple-negative breast cancers. Cell Cycle.

[40] Bertero T, Oldham WM, Grasset EM, Bourget I, Boulter E, Pisano S (2019). Tumor-Stroma Mechanics Coordinate Amino Acid Availability to Sustain Tumor Growth and Malignancy. Cell Metab.

[41] Leone RD, Zhao L, Englert JM, Sun IM, Oh MH, Sun IH (2019). Glutamine blockade induces divergent metabolic programs to overcome tumor immune evasion. Science.

[42] Wang Y, Bai C, Ruan Y, Liu M, Chu Q, Qiu L (2019). Coordinative metabolism of glutamine carbon and nitrogen in proliferating cancer cells under hypoxia. Nat Commun.

[43] Bose S, Le A (2018). Glucose Metabolism in Cancer. Adv Exp Med Biol.

[44] Yang L, Achreja A, Yeung TL, Mangala LS, Jiang D, Han C (2016). Targeting Stromal Glutamine Synthetase in Tumors Disrupts Tumor Microenvironment-Regulated Cancer Cell Growth. Cell Metab.

[45] Mestre-Farrera A, Bruch-Oms M, Peña R, Rodríguez-Morató J, Alba-Castellón L, Comerma L (2021). Glutamine-Directed Migration of Cancer-Activated Fibroblasts Facilitates Epithelial Tumor Invasion. Cancer Res.

[46] Linares JF, Cordes T, Duran A, Reina-Campos M, Valencia T, Ahn CS (2017). ATF4-Induced Metabolic Reprograming Is a Synthetic Vulnerability of the p62-Deficient Tumor Stroma. Cell Metab.

[47] Olzmann JA, Carvalho P (2019). Dynamics and functions of lipid droplets. Nat Rev Mol Cell Biol.

[48] Koundouros N, Poulogiannis G (2020). Reprogramming of fatty acid metabolism in cancer. Br J Cancer.

[49] Munir R, Lisec J, Swinnen JV, Zaidi N (2019). Lipid metabolism in cancer cells under metabolic stress. Br J Cancer.

[50] Auciello FR, Bulusu V, Oon C, Tait-Mulder J, Berry M, Bhattacharyya S (2019). A Stromal Lysolipid-Autotaxin Signaling Axis Promotes Pancreatic Tumor Progression. Cancer Discov.

[51] Radhakrishnan R, Ha JH, Jayaraman M, Liu J, Moxley KM, Isidoro C (2019). Ovarian cancer cell-derived lysophosphatidic acid induces glycolytic shift and cancer-associated fibroblast-phenotype in normal and peritumoral fibroblasts. Cancer Lett.

[52] Kaffe E, Magkrioti C, Aidinis V (2019). Deregulated Lysophosphatidic Acid Metabolism and Signaling in Liver Cancer. Cancers (Basel).

[53] Gong J, Lin Y, Zhang H, Liu C, Cheng Z, Yang X (2020). reprogramming of lipid metabolism in cancer-associated fibroblasts potentiates migration of colorectal cancer cells. Cell Death Dis.

[54] Sousa CM, Biancur DE, Wang X, Halbrook CJ, Sherman MH, Zhang L (2016). Pancreatic stellate cells support tumour metabolism through autophagic alanine secretion. Nature.

[55] Mishra R, Haldar S, Placencio V, Madhav A, Rohena-Rivera K, Agarwal P (2018). Stromal epigenetic alterations drive metabolic and neuroendocrine prostate cancer reprogramming. J Clin Invest.

[56] Yang Y, Yang X, Yang Y, Zhu H, Chen X, Zhang H (2015). Exosomes: A Promising Factor Involved in Cancer Hypoxic Microenvironments. Curr Med Chem.

[57] Ruivo CF, Adem B, Silva M, Melo SA (2017). The Biology of Cancer Exosomes: Insights and New Perspectives. Cancer Res.

[58] Tkach M, Théry C (2016). Communication by Extracellular Vesicles: Where We Are and Where We Need to Go. Cell.

[59] Gangoda L, Boukouris S, Liem M, Kalra H, Mathivanan S (2015). Extracellular vesicles, including exosomes are mediators of signal transduction: are they protective or pathogenic?. Proteomics.

[60] Yang X, Li Y, Zou L, Zhu Z (2019). Role of Exosomes in Crosstalk Between Cancer-Associated Fibroblasts and Cancer Cells. Front Oncol.

[61] Zhao H, Yang L, Baddour J, Achreja A, Bernard V, Moss T (2016). Tumor microenvironment derived exosomes pleiotropically modulate cancer cell metabolism. Elife.

[62] Achreja A, Zhao H, Yang L, Yun TH, Marini J, Nagrath D (2017). Exo-MFA-A 13C metabolic flux analysis framework to dissect tumor microenvironment-secreted exosome contributions toward cancer cell metabolism. Metab Eng.

[63] Kunou S, Shimada K, Takai M, Sakamoto A, Aoki T, Hikita T (2021). Exosomes secreted from cancer-associated fibroblasts elicit anti-pyrimidine drug resistance through modulation of its transporter in malignant lymphoma. Oncogene.

[64] Li Y, Zhao Z, Liu W, Li X (2020). SNHG3 Functions as miRNA Sponge to Promote Breast Cancer Cells Growth Through the Metabolic Reprogramming. Appl Biochem Biotechnol.

[65] Ahn CS, Metallo CM (2015). Mitochondria as biosynthetic factories for cancer proliferation. Cancer Metab.

[66] Zhang Z, Gao Z, Rajthala S, Sapkota D, Dongre H, Parajuli H (2020). Metabolic reprogramming of normal oral fibroblasts correlated with increased glycolytic metabolism of oral squamous cell carcinoma and precedes their activation into carcinoma associated fibroblasts. Cell Mol Life Sci.

[67] Ippolito L, Morandi A, Taddei ML, Parri M, Comito G, Iscaro A (2019). Cancer-associated fibroblasts promote prostate cancer malignancy via metabolic rewiring and mitochondrial transfer. Oncogene.

[68] Sansone P, Savini C, Kurelac I, Chang Q, Amato LB, Strillacci A (2017). Packaging and transfer of mitochondrial DNA via exosomes regulate escape from dormancy in hormonal therapy-resistant breast cancer. Proc Natl Acad Sci U S A.

[69] Sun L, Sun C, Liang Z, Li H, Chen L, Luo H (2015). FSP1(+) fibroblast subpopulation is essential for the maintenance and regeneration of medullary thymic epithelial cells. Sci Rep.

[70] Curtis M, Kenny HA, Ashcroft B, Mukherjee A, Johnson A, Zhang Y (2019). Fibroblasts Mobilize Tumor Cell Glycogen to Promote Proliferation and Metastasis. Cell Metab.

[71] Zhao L, Ji G, Le X, Wang C, Xu L, Feng M (2017). Long Noncoding RNA LINC00092 Acts in Cancer-Associated Fibroblasts to Drive Glycolysis and Progression of Ovarian Cancer. Cancer Res.

[72] Kumar D, New J, Vishwakarma V, Joshi R, Enders J, Lin F (2018). Cancer-Associated Fibroblasts Drive Glycolysis in a Targetable Signaling Loop Implicated in Head and Neck Squamous Cell Carcinoma Progression. Cancer Res.

[73] Demircioglu F, Wang J, Candido J, Costa ASH, Casado P, de Luxan Delgado B (2020). Cancer associated fibroblast FAK regulates malignant cell metabolism. Nat Commun.

[74] Tommelein J, De Vlieghere E, Verset L, Melsens E, Leenders J, Descamps B (2018). Radiotherapy-Activated Cancer-Associated Fibroblasts Promote Tumor Progression through Paracrine IGF1R Activation. Cancer Res.

[75] Broekgaarden M, Anbil S, Bulin AL, Obaid G, Mai Z, Baglo Y (2019). Modulation of redox metabolism negates cancer-associated fibroblasts-induced treatment resistance in a heterotypic 3D culture platform of pancreatic cancer. Biomaterials.

[76] Chan JS, Tan MJ, Sng MK, Teo Z, Phua T, Choo CC (2017). Cancer-associated fibroblasts enact field cancerization by promoting extratumoral oxidative stress. Cell Death Dis.

[77] Cheteh EH, Augsten M, Rundqvist H, Bianchi J, Sarne V, Egevad L (2017). Human cancer-associated fibroblasts enhance glutathione levels and antagonize drug-induced prostate cancer cell death. Cell Death Dis.

[78] Oudin MJ, Weaver VM (2016). Physical and Chemical Gradients in the Tumor Microenvironment Regulate Tumor Cell Invasion, Migration, and Metastasis. Cold Spring Harb Symp Quant Biol.

[79] Grassian AR, Coloff JL, Brugge JS (2011). Extracellular matrix regulation of metabolism and implications for tumorigenesis. Cold Spring Harb Symp Quant Biol.

[80] Pickup MW, Mouw JK, Weaver VM (2014). The extracellular matrix modulates the hallmarks of cancer. EMBO Rep.

[81] Zhang J, Chen L, Liu X, Kammertoens T, Blankenstein T, Qin Z (2013). Fibroblast-specific protein 1/S100A4-positive cells prevent carcinoma through collagen production and encapsulation of carcinogens. Cancer Res.

[82] Liu QP, Luo Q, Deng B, Ju Y, Song GB (2020). Stiffer Matrix Accelerates Migration of Hepatocellular Carcinoma Cells through Enhanced Aerobic Glycolysis Via the MAPK-YAP Signaling. Cancers (Basel).

[83] Petruzzelli M, Wagner EF (2016). Mechanisms of metabolic dysfunction in cancer-associated cachexia. Genes Dev.

[84] Lok C (2015). Cachexia: The last illness. Nature.

[85] Fearon KC, Glass DJ, Guttridge DC (2012). Cancer cachexia: mediators, signaling, and metabolic pathways. Cell Metab.

[86] Patel HJ, Patel BM (2017). TNF-α and cancer cachexia: Molecular insights and clinical implications. Life Sci.

[87] Garin-Chesa P, Old LJ, Rettig WJ (1990). Cell surface glycoprotein of reactive stromal fibroblasts as a potential antibody target in human epithelial cancers. Proc Natl Acad Sci U S A.

[88] Levy MT, McCaughan GW, Abbott CA, Park JE, Cunningham AM, Müller E (1999). Fibroblast activation protein: a cell surface dipeptidyl peptidase and gelatinase expressed by stellate cells at the tissue remodelling interface in human cirrhosis. Hepatology.

[89] Brokopp CE, Schoenauer R, Richards P, Bauer S, Lohmann C, Emmert MY (2011). Fibroblast activation protein is induced by inflammation and degrades type I collagen in thin-cap fibroatheromata. Eur Heart J.

[90] Bauer S, Jendro MC, Wadle A, Kleber S, Stenner F, Dinser R (2006). Fibroblast activation protein is expressed by rheumatoid myofibroblast-like synoviocytes. Arthritis Res Ther.

[91] Yang X, Lin Y, Shi Y, Li B, Liu W, Yin W (2016). FAP Promotes Immunosuppression by Cancer-Associated Fibroblasts in the Tumor Microenvironment via STAT3-CCL2 Signaling. Cancer Res.

[92] Roberts EW, Deonarine A, Jones JO, Denton AE, Feig C, Lyons SK (2013). Depletion of stromal cells expressing fibroblast activation protein-α from skeletal muscle and bone marrow results in cachexia and anemia. J Exp Med.

[93] Daou HN (2020). Exercise as an anti-inflammatory therapy for cancer cachexia: a focus on interleukin-6 regulation. Am J Physiol Regul Integr Comp Physiol.

[94] Han J, Meng Q, Shen L, Wu G (2018). Interleukin-6 induces fat loss in cancer cachexia by promoting white adipose tissue lipolysis and browning. Lipids Health Dis.

[95] Flint TR, Janowitz T, Connell CM, Roberts EW, Denton AE, Coll AP (2016). Tumor-Induced IL-6 Reprograms Host Metabolism to Suppress Anti-tumor Immunity. Cell Metab.

[96] Argilés JM, Busquets S, López-Soriano FJ (2003). Cytokines in the pathogenesis of cancer cachexia. Curr Opin Clin Nutr Metab Care.

[97] Qin X, Yan M, Wang X, Xu Q, Wang X, Zhu X (2018). Cancer-associated Fibroblast-derived IL-6 Promotes Head and Neck Cancer Progression via the Osteopontin-NF-kappa B Signaling Pathway. Theranostics.

[98] Ebbing EA, van der Zalm AP, Steins A, Creemers A, Hermsen S, Rentenaar R (2019). Stromal-derived interleukin 6 drives epithelial-to-mesenchymal transition and therapy resistance in esophageal adenocarcinoma. Proc Natl Acad Sci U S A.

[99] Heichler C, Scheibe K, Schmied A, Geppert CI, Schmid B, Wirtz S (2020). STAT3 activation through IL-6/IL-11 in cancer-associated fibroblasts promotes colorectal tumour development and correlates with poor prognosis. Gut.

[100] Lee JJ, Perera RM, Wang H, Wu DC, Liu XS, Han S (2014). Stromal response to Hedgehog signaling restrains pancreatic cancer progression. Proc Natl Acad Sci U S A.

[101] Rhim AD, Oberstein PE, Thomas DH, Mirek ET, Palermo CF, Sastra SA (2014). Stromal elements act to restrain, rather than support, pancreatic ductal adenocarcinoma. Cancer Cell.

[102] Özdemir BC, Pentcheva-Hoang T, Carstens JL, Zheng X, Wu CC, Simpson TR (2014). Depletion of carcinoma-associated fibroblasts and fibrosis induces immunosuppression and accelerates pancreas cancer with reduced survival. Cancer Cell.

[103] Wu D, Zhuo L, Wang X (2017). Metabolic reprogramming of carcinoma-associated fibroblasts and its impact on metabolic heterogeneity of tumors. Semin Cell Dev Biol.

[104] Mimura I, Nangaku M, Kanki Y, Tsutsumi S, Inoue T, Kohro T (2012). Dynamic change of chromatin conformation in response to hypoxia enhances the expression of GLUT3 (SLC2A3) by cooperative interaction of hypoxia-inducible factor 1 and KDM3A. Mol Cell Biol.

[105] Branco MR, Ficz G, Reik W (2011). Uncovering the role of 5-hydroxymethylcytosine in the epigenome. Nat Rev Genet.

[106] LeBleu VS, Neilson EG (2020). Origin and functional heterogeneity of fibroblasts. Faseb j.

[107] Najafi M, Mortezaee K, Majidpoor J (2019). Stromal reprogramming: A target for tumor therapy. Life Sci.

[108] Bu L, Baba H, Yasuda T, Uchihara T, Ishimoto T (2020). Functional diversity of cancer-associated fibroblasts in modulating drug resistance. Cancer Sci.

